# Long term outcomes of cataract surgery in severe and end stage primary angle closure glaucoma with controlled IOP: a retrospective study

**DOI:** 10.1186/s12886-020-01434-9

**Published:** 2020-04-19

**Authors:** Lin Fu, Yau Kei Chan, Junhua Li, Li Nie, Na Li, Weihua Pan

**Affiliations:** 1 Department of Glaucoma, Affiliated Eye Hospital, School of Ophthalmology and Optometry, Wenzhou Medical University, 618# Feng Qi Dong Road, Hangzhou Zhejiang Province, 325000 People’s Republic of China; 2 Department of Ophthalmology, LKS Faculty of Medicine, University of Hong Kong, Pok Fu Lam, Hong Kong SAR

**Keywords:** Cataract extraction, Severe and end stage of glaucoma, Visual acuity, Intraocular pressure

## Abstract

**Background:**

To investigate the long term surgical outcomes of cataract surgery in severe and end stage glaucoma patients with preoperative intraocular pressure less than 21 mmHg, and to detect the associated factors.

**Methods:**

A retrospective study of primary angle closure glaucoma patients was conducted on who underwent cataract surgery or combined with goniosynechialysis from March 2015 to April 2018. Main outcome measures were visual acuity, intraocular pressure, number of glaucoma medications and complications.

**Results:**

Sixteen patients (19 eyes) were included. The mean age was 64.89 ± 11.68 years and the mean followed up duration was 21.89 ± 7.85 months. The final visual acuity was significantly improved from 0.69 ± 0.55 to 0.46 ± 0.52 logMAR, within 12 (63.2%) eyes improved, 4 (21.1%) eyes kept unchanged, and 3 (15.8%) eyes reduced. Linear regression analysis indicated that higher mean deviation, higher visual field index and lower glaucoma stage associated with better final visual acuity (*r* = − 0.511, *r* = − 0.493, *r* = 0.537 respectively). Moreover, the final number of medications were reduced from 1.26 ± 0.99 to 0.26 ± 0.56 (*p* < 0.01). The mean intraocular pressure was not significantly reduced with the final IOP of 14.48 ± 3.74 mmHg (*p* = 0.97). While the eyes with intraocular pressure above 15 mmHg was decreased to 6 (31.6%) eyes compared to 10 (52.6%) eyes at baseline. Moreover, the number of eyes free of medications was increased from 4 (21.1%) preoperatively to 15 (78.9%) eyes postoperatively.

**Conclusions:**

Final visual acuity was significantly improved in the severe and end stage primary angle closure glaucoma patients and the number of eyes came off medications increased by 57.8% after cataract surgery. Preoperatively, the glaucoma stage, mean deviation and visual field index are important parameters to predict the final visual acuity after cataract surgery.

## Background

Glaucoma and cataract constitute the top two leading causes of blindness worldwide which significantly affect the visual impairment and quality of life [1, 2]. They are commonly coexisted as both are age-related [3, 4]. Their relative impact on the visual function are difficult to differentiate when they are coexisting. Meanwhile, cataract can have a great influence on quality of life in glaucoma patients with differed severities [5].

However, in severe and end stage glaucoma, postoperative “wipe out” is a risky complication. The “wipe out” is defined as a sudden visual acuity (VA) loss without apparent reasons especially in advanced glaucoma after filtering surgery [6, 7]. Moreover, eyes with glaucoma are at increased risk of complications like posterior capsular tear with vitrectomy, postoperative inflammation, prolonged increase of intraocular pressure (IOP) and achieve less significant visual improvement than eyes without glaucoma after cataract surgery [8]. Accordingly, only patients with medically uncontrolled glaucoma may warrant a surgery. Conservative managements, instead of cataract surgery, are much preferred for glaucoma patients with controlled IOP.

Glaucomatous eyes, as reported, can still gain satisfactory visual outcomes after phacoemulsification [8]. Combined phacoemulsification and non-penetrating deep sclerectomy (NPDS) was ever performed in the severe and end stage glaucoma patients, no “wipe out” was observed and the mean VA was improved at month-6 postoperatively [9]. Another study demonstrated that 73% glaucoma patients with low vision had better vision or maintained at 5 years after cataract surgery [10]. The improved visual function like VA and visual field index, is suggested to be related with better quality of life in glaucoma patients [11]. Therefore, it is advocated that cataract surgery should be offered in glaucoma patients who are IOP controlled. Cataract extraction not only improves the visual function, but also reduces the IOP [12, 13]. Due to the preservatives in glaucoma medications like benzalkonium chloride (BAK) and sodium perborate, longer treatment period and the use of multiple glaucoma drugs, ocular surface diseases (OSD) are more common in glaucomatous eyes using topical medications than glaucomatous eyes without medications and also normal eyes [14–16]. The IOP lowering effect of cataract surgery can reduce the usage of glaucoma medications, thereby alleviate the OSD and improve patient quality of life. Hence, despite the potential risky complications, cataract surgery is still recommended for severe and end stage glaucoma patients with IOP controlled to improve their life quality.

In previous studies, patients who were defined as medically uncontrolled were with IOP higher than 21 mmHg or with the use of more than 3 glaucoma medications [17, 18]. Here, the controlled IOP was defined as IOP lower than 21 mmHg, and requiring not more than 3 topical glaucoma drugs. To provide better predication on the VA outcome in severe and end stage glaucoma with controlled IOP, in this work, we retrospectively studied the outcomes of cataract surgery of these patients in primary angle closure glaucoma (PACG). The long-term VA outcome, as well as the predictive factors of final VA in these patients, are studied and reported here.

## Methods

### Patients and study design

A retrospective study was conducted to review the medical charts from the high risk surgery bank in our hospital. Primary angle closure glaucoma patients in the severe and end stage who underwent cataract surgery from March 2015 to April 2018 were retrieved. The investigational study was approved by the Institutional Review Board of the Wenzhou Medical University and in accordance with the tenets of the Declaration of Helsinki.

The diagnosis of PACG was referred to the previous criteria reported by Husain [19]. PACG was identified when posterior pigmented trabecular meshwork was not observed for at least 180 on gonioscopy without indentation in the primary position of gaze, as well as the presence of glaucomatous optic nerve damage and visual field defect on perimetry. Referred to the modified Bascom Palmer Glaucoma Staging System, severe glaucoma (stage 4) was confirmed when a mean deviation (MD) < − 20 dB and one of the following three criteria was met by the 30–2 Humphrey perimetry preoperatively: 1. on pattern deviation plot, 50 to 75% points depressed below the 5% level or 25 to 50% points depressed below the 1% level; 2. there were more than 1 points with sensitivity of 0 dB in the central 5° area; 3. at least one point with sensitivity of less than 15 dB in both hemifields within 5° of fixation. The end stage glaucoma (stage 5) was defined by the VA < 20/200 or unavailable to perform the Humphrey visual field examination attributable to glaucoma [19]. For the purpose of statistical analysis, the MD of stage 5 eyes were considered as − 33 dB and the visual field index (VFI) of these eyes were 0%.

The inclusion criteria were: 1. older than 30 years; 2. diagnosis of PACG with stage 4 and stage 5 severity; 3. sufficient lens opacity to induce vision reduction evaluated by the operating surgeon; 4. with a follow-up of at least 1 year; 5. preoperative IOP was less than 21 mmHg. The exclusion criteria were: 1. complicated with other ocular disorders affecting the visual acuity: corneal opacity, lens dislocation, diabetic retinopathy and ischemic optic neuropathy; 2. incomplete set of required data.

### Preoperative and postoperative examination

Preoperatively, the following information was collected for each eye: age, gender, glaucoma type, cataract grading (LOCSII) vertical cup to disc ratio, gonioscopy, visual field results, IOP, number of glaucoma medication, VA, best corrected visual acuity (BCVA), previous surgical history including filtration surgery and laser peripheral iridotomy (LPI). After surgery, the VA, IOP, glaucoma medication at the first month, 6th month and final visit, complication and intervention was recorded. The case of study was considered as “wipe out” if the postoperative VA reduced to < 20/200, or to counting fingers or less when preoperative VA was < 20/200. The VA and BCVA was measured as decimal units and converted into a logarithm of the minimum angle of resolution (logMAR). For the purpose of statistical analysis, counting fingers and hand motion were equating to 1/200 and 0.5/200 [20].

### Surgical technique

Phacoemulsification and intraocular lens (IOL) implantation (PEI) was carried out in these eyes. Briefly, a main and lateral corneal incision, continuous curvilinear capsulorrhexis, hydrodissection, phacoemulsification, residual cortex removal, a foldable IOL implantation in the capsular bag were performed. If peripheral anterior synechia (PAS) was observed under gonioscope in the preoperative examination, combined PEI and goniosynechialysis (PEI-GSL) was conducted then. The viscoelastic was injected to the anterior chamber to separate the PAS. If the PAS was not opened, an iris repositor or similar instrument was applied to mechanically break the PAS. All the operations were performed by the same experienced surgeon (WH Pan).

### Statistical analysis

The data were analyzed in Prism 7 (GraphPad Software, Inc., San Diego, CA).. The numeric parameters were evaluated by Kolmogorov–Smirnov test for the distribution of normality. Kruskal-Wallis test was used to evaluate the categorical parameters. Friedman or one-way analysis of variance (ANOVA) tests was used to compare the quantitative variables. The factors related to VA changes were analyzed by Pearson or Spearman’s correlation. They are presented as mean ± standard deviations (SD) and range. Statistical significance was set at a *p* value of less than 0.05.

## Results

From the high risk surgery bank in our hospital from March 2015 to April 2018, 109 glaucoma patients underwent cataract surgery were reviewed. In total, 19 eyes in 16 patients with severe and end stage glaucoma were included for analysis. Table 1 summarized the baseline characteristics of the patients. Of the 19 PACG eyes, the mean age was 64.89 ± 11.68 (range, 44–80) years old. They followed up for 21.89 ± 7.85 (range, 12–39) months. Except phacoemulsification and cataract extraction, 17 eyes of them were underwent combined PEI-GSL. The mean number of preoperative medications were 1.26 ± 0.99 (range, 0–3) with only 6 (25%) eyesfree of medications and the mean IOP was 14.04 ± 3.49 (range, 8.1–17.9) mmHg. According to the modified Bascom Palmer Glaucoma Staging System, 16 (84.2%) and 3 (15.8%) of the eyes were stage 4 and 5 respectively. The mean MD was − 28.69 ± 3.27 dB (range, −33to − 21.4) and the mean VFI was 14.0% ± 10.95% (range, 0–37%).

**Table 1:** Demographic characteristics

	n (%) or mean ± SD (range)
Total Eyes	19
Age, years	64.89 ± 11.68 (44 to 80)
Male	10 (52.6%)
Right eyes	10 (52.6%)
Follow up, months	21.89 ± 7.85 (12 to 39)
Axial Length, mm	22.59 ± 0.85 (21.1 to 24.31)
PAS, degree	189.5 ± 108.8 (0 to 360)
Cataract degree	
Cortical	1.90 ± 0.74 (1 to 4)
Nuclear	1.58 ± 0.77 (0 to 3)
Posterior subcapsular	1.26 ± 0.65 (0 to 3)
Previous filtering surgery	2 (10.5%)
Previous LPI	10 (52.6%)
PEI-GSL	17 (89.5%)
Preoperative medication	1.26 ± 0.99 (0 to 3)
Preoperative IOP, mmHg	14.04 ± 3.49 (8.1–17.9)
Baseline VA, logMAR	0.69 ± 0.55 (0.1 to 2.6)
Baseline BCVA, logMAR	0.45 ± 0.59 (0 to 2.6)
Vertical cup disc ratio	0.94 ± 0.10 (0.7 to 1)
Stage 4	16 (84.2%)
Stage 5	3 (15.8%)
MD, dB	−28.69 ± 3.27 (− 33 to − 21.4)
VFI, %	14 ± 10.95 (0–37)

*SD* Standard deviation; *PAS* Peripheral anterior synechia; *LPI* Laser peripheral iridotomy; *PEI-GSL* Combined phacoemulsification, intraocular lens implantation and goniosynechialysis; *PACG* Primary angle closure glaucoma; *IOP* Intraocular pressure; *VA* Visual acuity; *logMAR* Logarithm of the minimum angle of resolution; *BCVA* Best corrected visual acuity; *MD* Mean deviation, *dB* Decibel, *VFI* Visual field index

**Fig. 1:**
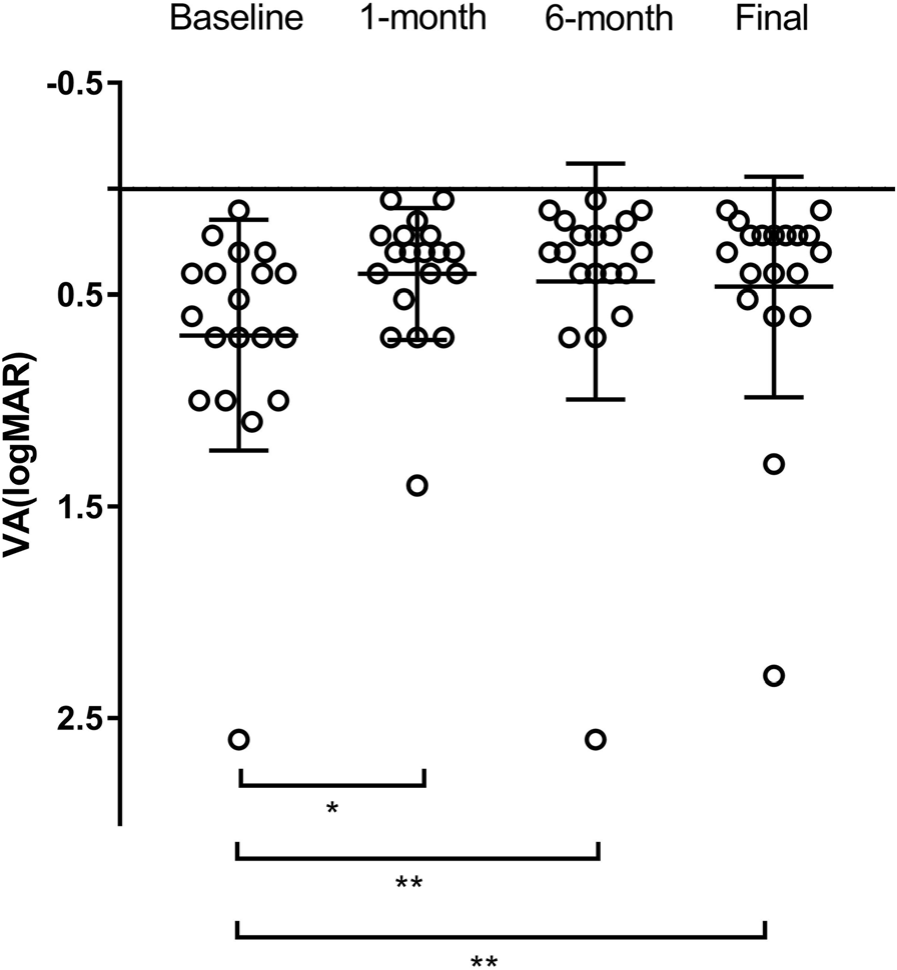
Changes in preoperative visual acuity (VA) and postoperative VA. Postoperative VA levels were all significantly improved compared with preoperative VA at 1 month, 6 months and final visit. *p < 0.05 and **p < 0.01. logMAR = logarithm of the minimum angle of resolution. The patient number was 19 in all the visits

**Fig. 2:**
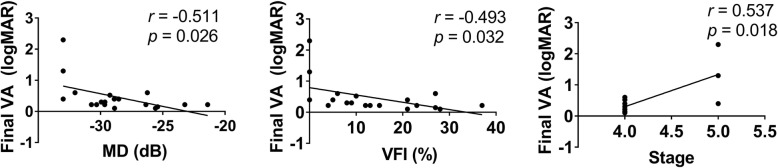
Scatter plots showing factors related to final visual acuity (VA). logMAR = logarithm of the minimum angle of resolution; MD = mean deviation, dB = decibel, VFI = visual field index

Changes in baseline and postoperative VA are shown in Fig. 1. The mean VA at baseline was 0.69 ± 0.55 (range, 0.1 to 2.6) logMAR unit. The VA levels were significantly improved in all the postoperative visits at 1 month, 6 months and the final visit (*p* < 0.05, *p* < 0.01 and *p* < 0.01). They were 0.40 ± 0.31 (range, 0.05 to 1.4), 0.43 ± 0.31 (range, 0.05 to 2.6) and 0.46 ± 0.52 (range, 0.1 to 2.3) logMAR unit respectively. The percentage of postoperative VA improvement, unchanged and reduction are listed in Table 2. Final VA improved in 12 (63.2%) eyes and was unchanged in 4 (21.1%) eyes (Table 2). Moreover, no cases of “wipe out” was detected. Linear regression was performed to analyze the factors associated with the final VA. The baseline MD, VFI and glaucoma stage were found to be related to the final VA (*r* = − 0.511, *p* = 0.026; *r* = − 0.493, *p* = 0.032; *r* = 0.537, *p* = 0.018) (Fig. 2). These indicated that higher MD, higher VFI and lower glaucoma stage indicated better final VA. For the 3 eyes in the end stage, the baseline VA was 1.0, 2.6 and 0.4 logMAR unit. And the final VA of them were 1.3, 2.3 and 0.4 logMAR unit accordingly. This means 1 eye was reduced, 1 eye was improved and the left 1 eye was unchanged.

**Table 2:** VA changes in postoperative visits

	Improved, n(%)	unchanged, n(%)	worsen, n(%)
1-month	17 (89.5%)	0 (0%)	2 (10.5%)
6-month	14 (73.7%)	4 (21.1%)	1 (5.3%)
Final	12 (63.2%)	4 (21.1%)	3 (15.8%)

*VA* Visual acuity

**Fig. 3:**
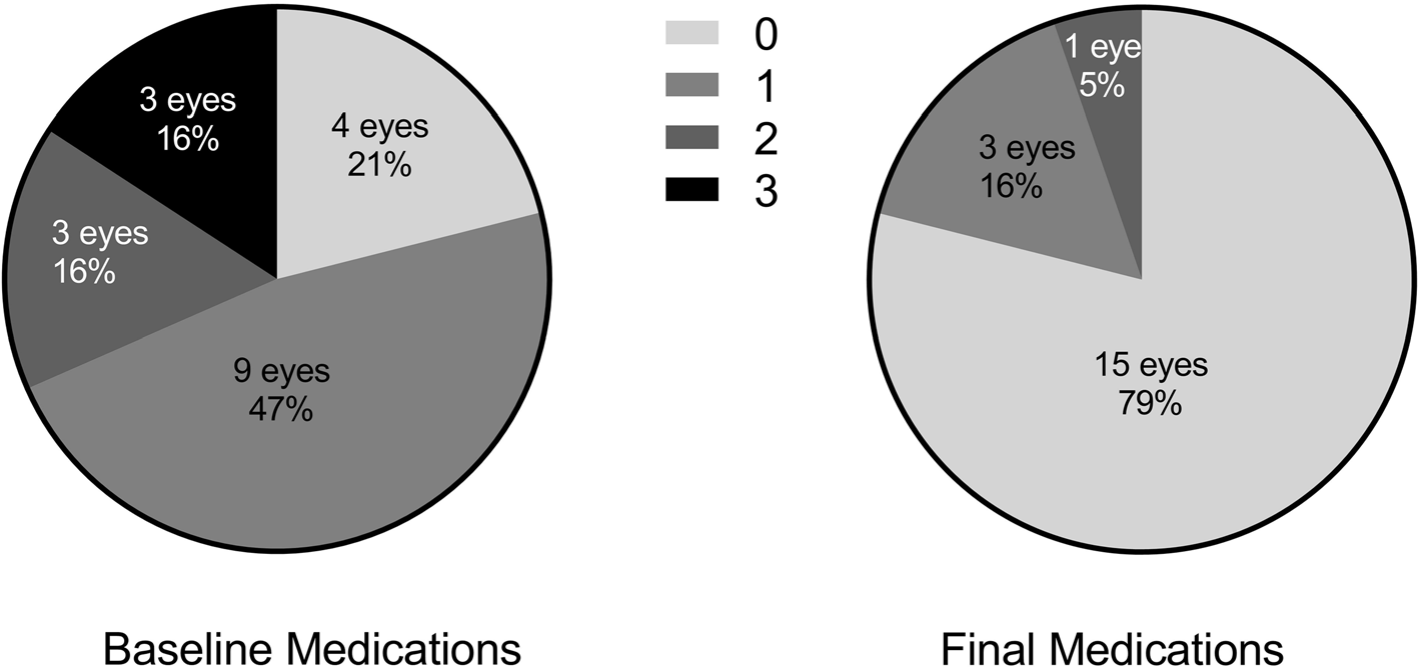
The baseline and final number of medications

The final number of antiglaucoma medications were significantly reduced from 1.26 ± 0.99 (range, 0–3) to 0.26 ± 0.56 (range, 0–2) (*p* < 0.01). The number of eyes that came off medications improved from preoperatively 4 (21.1%) eyes to 15 (78.9%) eyes postoperatively (Fig. 3). The number of eyes with IOP above 15 mmHg at baseline were 10 (52.6%) and was reduced to 6 (31.6%) at the final visit, although the mean IOP between the baseline and final was not statistically different with the final IOP of 14.48 ± 3.74 (9.9–25.9) mmHg (*p* > 0.05). There was 9 cases of IOP increased at the final visit with 8 of them free of medication and 1 was on 1 glaucoma medication. All the IOP values were under 21 mmHg except 1 eye was 25.9 mmHg, while his final VA was 0.4 logMAR which was higher than the baseline VA of 0.7 logMAR.

The postoperative complications are shown in Table 3. The incidence of posterior capsular opacity (PCO) was 10.5% and was only observed in the VA improved group within 2 eyes. The time of the PCO detected was 31 months and 12 months after surgery in these 2 eyes respectively. All of them were underwent Neodymium: YAG laser posterior capsulotomy immediately. Shallow anterior chamber was found in 1 eye at 12 months after surgery in the VA unchanged group and the laser peripheral iridotomy was performed. Malignant glaucoma occurred in 1 eye of the VA unchanged group 5 days after operation. Anterior vitrectomy was performed and effectively managed this complication.

**Table 3:** Postoperative complications

	Improved group	Unchanged group	Worsen group
	(*n* = 12)	(*n* = 4)	(*n* = 3)
Posterior capsular opacity	2 (10.5%)		
Malignant glaucoma		1 (5.3%)	
Shallow anterior chamber		1 (5.3%)	

## Discussion

The information of the long term surgical outcomes in severe and end stage glaucoma with controlled IOP is scarce. Particularly, the understanding on the visual outcome of these patients after cataract surgery is lacking. The reported surgical outcomes of cataract surgery in severe and end stage glaucoma in literature were mainly come up from patients who were medically uncontrolled [9, 21]. In glaucoma patients with controlled IOP, cataract surgery is seldomly performed since they are at high risk of “wipe out” [6, 7]. Here, our present retrospective study instead shows that the postoperative VA of PACG patients significantly improved after cataract surgery. In addition, the number of glaucoma medications also significantly reduced. Moreover, the baseline MD, VFI and glaucoma stage may help to predict the visual outcome after surgery.

Cataract extraction was reported to improve the VA in glaucoma patients with most of them were in the early stage or medically uncontrolled. For instance, in the Collaborative Initial Glaucoma Treatment Study (CIGTS), the VA was improved abruptly after cataract extraction and maintained for 1.5 years in glaucoma with preoperative mean deviation of − 5.74 dB [22]. And in 2018, Igor et al. reported that the VA of severe and end stage glaucoma patients was not improved when combined the glaucoma surgery of NPDS with phacoemulsification [23]. While in his later study, the VA was improved after the same surgeries [9]. Both of the studies were last for 6 months. The difference in conclusion may be due to the difference in sample sizes, with only 5 in the earlier one study and then increased to 18 eyes for the later. Even in these medically uncontrolled eyes, VA can be improved after cataract surgery although the primary purpose was to reduce IOP. We hence speculated that cataract extraction can also improve VA in IOP controlled eyes after surgery in these severe and end stage glaucoma. In clinical settings, IOP is always the primary focus in glaucoma management while VA is rarely considered as an assessment parameter of treatment outcome in glaucoma patients. However, VA is highly reflecting the life quality of patients. For glaucoma patients in the severe and end stage with constricted visual field, VA indeed reflects more about the subject perception and the ability to interact with environment [10]. Accordingly, VA improvement in these patients offer an important opportunity to improve their quality of life. Here, we pioneeringly provided the information that the mean VA improved from 0.69 ± 0.55 to 0.46 ± 0.52 logMAR unit with a mean follow up of 21.89 ± 7.85 months, in IOP controlled severe and end stage PACG patients. This result is encouraging and useful since little is known about the visual outcome of severe and end stage glaucoma patients with IOP controlled before. It is difficult to quantify the vision reduction contributed by cataract or glaucoma independently but based on this study we can now suggest VA can be improved in these patients after cataract surgery.

The reason that cataract surgery was seldom performed solely for the purpose of visual improvement in severe and end stage glaucoma patients with controlled IOP, is due to the risk of “wipe out”. It is a long-standing debate whether cataract surgery should be performed on patients with severe and end stage glaucoma. In the past, the reported incidence of “wipe out” in end stage glaucoma was discrepant. Some suggest this to be a rare or even non-existent complication and others fear the risk of sudden visual loss [6, 21, 24]. In our study, no cases of “wipe out” occurred. Most of the studies that reported high rate of “wipe out” were more than 26 years ago. Nowadays, with the advanced technologies, complications can be well resolved and “wipe out” might have a chance to be relegated to a place in history [25]. “Wipe out” was regarded as a sudden vision loss without apparent causes especially in advanced glaucoma after filtering surgery [6, 7], and was suspected to be related with ocular hypotony during surgery. In this study, all the surgeries were performed by a single experienced glaucoma specialist, no cases of “wipe out” was observed and 84.2% eyes showed better or maintained VA at the final visit. These results therefore supported that cataract surgery in general is safe and effective on patients with severe and end stage glaucoma patients. For the eyes with postoperative complications, 2 eyes had PCO and were managed by posterior capsulectomy and their final VA were improved. Malignant glaucoma occurred in one eye and shallow anterior chamber happened in another eye. Both showed unchanged VA in the final checking. In addition, from our linear regression analysis, the greater baseline MD, higher VFI and lower glaucoma stage may predict better VA after cataract surgery. However, VA is not directly related to the visual field. The functional visual acuity (FVA) measured by an AS-28 FVA measurement system had shown a weak correlation with MD in glaucoma with different severities [26]. Here, we hypothesize that the VA may be more associated with visual filed parameters in severely damaged glaucoma. To our knowledge, the visual field parameters and glaucoma stage have not been indicated as the predictive factors of the VA outcomes in severe and end stage glaucoma after cataract surgery. These factors may provide an important reference to the decision management of treatments for these patients.

Traditionally, in PACG, the surgical methods were compared between the PEI alone and combined phacotrabeculectomy. It was proved that combined phacotrabeculectomy was more effective in IOP lowering than PEI alone irrespective baseline IOP control. However, it had more postoperative complications (8 complications vs 0 complication) like “wipe out”, ocular hypotony and poor IOP control [17, 27]. Compared with trabeculectomy, GSL was reported to be safe with mild complications including intraoperative hyphema, mild zonulysis and postoperative IOP spikes [28, 29]. Medically uncontrolled glaucomatous eyes warrant surgeries to decease IOP since elevated IOP increases the risk of glaucoma progression. In medically controlled eyes, especially in the patients with constrict visual field, balancing the risk and benefit of surgery should be more careful. And GSL is more suitable than traditional procedure like trabeculectomy for patients with severely damaged optic nerve.

In the present study, 17 eyes underwent combined PEI-GSL and only 2 eyes received PEI alone since in the perioperative examination, PAS was not found in these 2 eyes. PEI alone was reported to sufficiently reduce the IOP in PACG and whether GSL should be combined was controversial in previous studies [29, 30]. The mechanism that PEI decreasing the IOP in PACG is that lens extraction can partly relieves the role of anteriorly positioned lens in the PACG by implanting a much thinner IOL. And PEI itself may mechanically open some PAS by the use of viscoelastic during procedure. The various effects of GSL may be due to the differed PAS, study population and short follow up duration. In the most recent one randomized clinical trial, GSL did not show additional IOP lowering effect over PEI alone [29]. However, the subgroups of Singapore and Vietnam displayed opposite results of the two surgical procedures. And this may be the reason that the final result did not demonstrate a significant difference between PEI and PEI-GSL. In addition, at 12 months after surgery, the PAS in the PEI-GSL was slight less than in the PEI in the Husain’s study [29]. This may indicate that combined with GSL may warrant a longer time for angle open in PACG which in turn will benefit the IOP maintainance. Hence, in our study, although the IOP was normal preoperatively, GSL was still performed in eyes with PAS. It is said higher baseline IOP resulted greater IOP reduction [29]. In this study, the preoperative IOP was all under 21 mmHg with a mean IOP of 13.8 ± 3.3 mmHg. And there was no significant change after surgery with a mean postoperative IOP of 13.2 ± 3.9 mmHg. Nevertheless, it can be noticed that percentage of eyes with IOP above 15 mmHg reduced from 52.6% at baseline to 31.6% at the final visit. And it can also be revealed on the other hand, the IOP control after cataract extraction was demonstrated by the decrease of topical glaucoma drugs used in the postoperative period. The number of glaucoma medications were significantly reduced. Also, the percentage of patients who came off topical glaucoma drugs was greatly increased from 21.1 to 78.9%. It would be meaningful to investigate how such reduction of the drugs impacts on the quality of life of patients, financial costs and adverse effect from the drugs.

Since this is a retrospective study, we mainly used the VA as the indicator of the success of cataract surgery. Other measures like subjective visual function, color perception and overall satisfaction to surgery were not documented. They may also be important parameters to evaluate the quality of life of patients. Also, due to the limited sample size and follow up duration, the current dataset is insufficient to analyze the significance of glaucomatous progression and related complications. A larger sample size of prospective study is needed for further justifications.

## Conclusions

In conclusion, cataract extraction provides an additional opportunity of VA improvement in severe and end stage glaucoma patients with controlled IOP. It may also relief the life burden of patients by reducing or even coming off their topical medications. The prediction of VA outcome can refer to the preoperative visual field parameters including MD, VFI and glaucoma stage. The results from our study may change the traditional management practice of severe and end stage glaucoma with controlled IOP and greatly improve the quality of life of patients.

## Data Availability

The datasets used and/or analyzed during the current study are available from the corresponding author on reasonable request.

## Abbreviations

VA: Visual acuity
IOP: Intraocular pressure
NPDS: Non-penetrating deep sclerectomy
BAK: Benzalkonium chloride
OSD: Ocular surface diseases
PACG: Primary angle closure glaucoma
MD: Mean deviation
VFI: Visual field index
BCVA: Best corrected visual acuity
LPI: Laser peripheral iridotomy
logMAR: Logarithm of the minimum angle of resolution
IOL: Intraocular lens
PEI: Phacoemulsification and intraocular lens implantation
PAS: Peripheral anterior synechia
PEI-GSL: Combined PEI and goniosynechialysis
ANOVA: One-way analysis of variance
SD: Standard deviations
PCO: Posterior capsular opacity
CIGTS: Collaborative initial glaucoma treatment study
FVA: Functional visual acuity
